# Effectiveness of behavioral sleep interventions on children’s and mothers’ sleep quality and maternal depression: a systematic review and meta-analysis

**DOI:** 10.1038/s41598-022-07762-8

**Published:** 2022-03-09

**Authors:** Jeongok Park, Soo Yeon Kim, Kyoungjin Lee

**Affiliations:** 1 Mo-Im Kim Nursing Research Institute, College of Nursing, Yonsei University, 616 College of Nursing, Yonsei-ro 50, Seodaemun-gu, Seoul, 03722 Korea; 2 College of Nursing and Brain Korea 21 FOUR Project, Yonsei University, Yonsei-ro 50, Seodaemun-gu, Seoul 03722 Korea

**Keywords:** Psychology and behaviour, Health care

## Abstract

This systematic review and meta-analysis was conducted to investigate the effectiveness of behavioral sleep interventions (BSIs) on the number of child night awakenings, and maternal sleep quality and depression. The search followed the Preferred Reporting Items for Systematic Reviews and Meta-Analyses guidelines (PRISMA) using PubMed, CINAHL, Cochrane, and EMBASE databases and retrieved studies published until April 2021. We calculated the odds ratios (ORs) and 95% confidence intervals (CIs) for child sleep problems, and the mean differences (MD) and 95% CI for the number of child night awakenings, and maternal sleep quality and depression. Ten studies of 1628 initial searched were included in the final analysis. Two of the 10 studies were divided into two subgroups by participants and intervention type; thus, 12 subgroups were included in the meta-analysis. BSIs significantly reduced child sleep problems (OR 0.51; 95% CI 0.37–0.69) and improved maternal sleep quality (MD − 1.30; 95% CI − 1.82 to − 0.77) in the intervention group. There were no significant differences in the number of child night awakenings and maternal depression between the two groups. More RCTs to examine the effect of BSIs considering children’s age, duration of intervention, and outcome measuring time points are needed.

## Introduction

Sleep problems are common in young children and occur in more than 20% of infants and toddlers^1,2^. These can adversely affect both children and mothers as the main caregivers of children. Sleep problems affect young children’s cognitive development^3–5^, behavior^3,6^, quality of life^7^, and place them at increased risk of obesity^8,9^. Previous studies found that 75% of children aged 0–4 years old had frequent night awakenings^10^, and infants who were short and persistent night-time sleepers could not integrate their sleep cycles until they were two or three years old^11^. Young children who slept for a short duration at night were five times more likely to show hyperactivity^12^. Young children’s sleep problems also affect their mothers’ physical and emotional health, and can cause insufficient maternal sleep duration, maternal fatigue, parenting stress, and depression^1,13–16^. These conditions can affect breastfeeding^13,15^, and lead to a lack of mother–child bonding, low parenting quality^14^, and poor development in children^17^.

The circadian rhythm is a natural internal process that regulates the sleep–wake pattern and repeats roughly every 24 h in humans^18^. Although this timing system is present during the fetal period, it is strongly influenced by the maternal circadian rhythm in the uterine environment^19^. At birth, being separated from the mother’s rhythm, the newborn will already possess an immature circadian rhythm, but this will still dramatically change over the first year of life^20^. The critical factor in developing an infant's immature 24-h circadian rhythm is the social interaction between mother and child^21^. For example, mothers provide fundamental care—from feeding to sleeping—to manage an infant’s daily routine by controlling the physical home environment day and night. However, these maternal roles are played in irregular sleep–wake patterns in mothers. The chief complaint of mothers in the 3–4-month postpartum period was fatigue due to irregular sleep–wake patterns as a result of caring for their babies^22^. Factors interfering with the establishment of regular circadian rhythms in children are associated with inconsistent interactions between parents and children and irregular sleep–wake patterns^23^. Therefore, behavioral sleep interventions (BSIs) that focus on establishing a stable circadian rhythm within the first year after birth known as critical periods are needed.

BSI for young children, such as settling and bedtime routines, are widely used training methods to improve sleep quality in children with sleep problems such as frequent night-time awakenings, short sleep duration, and sleep-onset latency. The settling methods are carried out in various ways, such as cry it out and fading^24,25^. The common effect of these methods is to allow children to self-soothe. These interventions provide guidance on sleep-related parenting behavior, such as delayed response to their baby’s signal or nocturnal cries, e.g.: extinction, graduated extinction methods under parental presence, encouraging self-soothing, and falling asleep independently. The settling intervention can help develop a child’s self-regulation abilities in preparation for the transition to a separate space without parental intervention. Another method to help children develop routine sleep habits is by regularly recognizing a bedtime^26,27^. Bedtime routines generally include sleep hygiene, such as bathing or relaxing massage. Other routines include regulating sleep, feeding, reading a book, singing a lullaby together, and other strategies to induce sleep in the baby. Previous studies reported that BSIs have resulted in a one-third reduction in sleep latency and fewer night awakenings—2.2 to 0.1 in the intervention group compared to the control group with no change^28^. These interventions also have been found to significantly improve sleep quality in parents^24–27^, children’s cognitive skills^29^, and parental mental health^24,30,31^. Furthermore, these BSIs can also be helpful for family functioning by reducing maternal distress and increasing marital satisfaction^32^.

Although a few systematic reviews (SR) related to the effectiveness of sleep interventions have been conducted, there were limitations, such as the lack of objectivity in synthesizing results, interventions being too broadly defined, or the small number of included studies. Thus, the effectiveness of BSIs for children remains unclear. For example, one SR^33^ did not fully follow the Preferred Reporting Items for SR and Meta-Analyses (PRISMA) guidelines^34^, regarding the selection process of the included studies and risk of bias in individual studies. The quality of assessment is an essential step in SR and can lead to adverse consequences if not appropriately performed^35^. Regarding the scope of the definition of sleep intervention, one SR included both a child sleep intervention and the mother’s psychosocial intervention (i.e., relaxation technique)^36^ and another SR study reported the synthesis of only two studies^37^.

Therefore, the purpose of this study was to evaluate behavioral sleep interventions for settling or bedtime routines of children aged 36 months or below. The goal was to identify the efficacy of BSIs in improving children’s sleep problems, improving maternal sleep quality and reducing depression, and reducing the frequency of children’s night awakenings.

## Materials and methods

### Protocol and registry

A SR and meta-analysis was performed according to the guidelines of the recently updated PRISMA guidelines^34^. The protocol for this SR was registered on PROSPERO (registration number: CRD42021229365) and is available on the PROSPERO website (https://www.crd.york.ac.uk/prospero/).

### Study design

This study was a SR and meta-analysis of randomized controlled trials (RCTs) to verify the effects of BSIs in children.

### Data sources and study selection

Electronic searches were conducted on the PubMed, Embase, CINHAL, and Cochrane databases from their inception to April 30, 2021.

The articles were selected in two steps. First, the titles and abstracts of the articles found through the search engines were reviewed by two independent researchers to eliminate articles that were not suitable for the current study. Second, the full text of the articles selected in the first step were then reviewed in order to obtain specific information on study design, participants, and interventions. Finally, the inclusion of studies that were originally disagreed upon by the researchers was determined through discussion.

### Search strategy

The database searches were limited to articles published in English, with human subjects, and which utilized RCTs by using the following Medical Subject Headings (MeSH) and keyword MeSH term and/or keywords in combinations: child, sleep, behavior, intervention, mothers, and depression. The search strategy is presented in detail in the Supplementary Table 1.

### Eligibility criteria

The subsequent inclusion criteria based on the PICO approach (population [P], intervention [I], comparators [C], outcome [O]) were used: (1) participants were young children aged 36 months or below; (2) interventions included BSIs based on self-settled, self-regulation, and bedtime routines; (3) comparators were the control groups who applied usual care and who did not receive BSIs; (4) outcome measures included child night awakenings, child sleep problems, maternal depression, and maternal sleep quality; and (5) RCTs designs published in a peer-reviewed journal and published in English. Exclusion criteria were (1) studies that were published in gray literature, such as abstracts and/or those that were not original articles, and (2) follow-up studies after an initial study.

### Definitions of intervention

BSIs were defined as follows: First, settling intervention (i.e., “self-settling” or “self-soothing” method) for child’s sleep was defined as a practice that aimed to focus on consolidating sleep and extending night sleep duration. Second, bedtime routine interventions was defined as a series of consistent and repetitive activities that was carried out before bedtime every night. These interventions have no standard components since they depend on the developmental stage of the children and the cultural situation. Therefore, bedtime routine interventions was defined as a practice that consisted of more than two activities before going to sleep.

### Outcome measures

All extracted outcome variables in the included studies are presented in detail in Supplementary Table 2. Studies that evaluated two specific aspects were chosen. The first aspect was child night awakenings and sleep problems reported by mothers using a sleep diary for their children. The second aspect was maternal sleep quality measured by the Pittsburgh Sleep Quality Index (PSQI), and maternal depression measured by the Edinburgh Postnatal Depression Scale (EPDS) for mothers.

### Quality of appraisal

The Risk of Bias 2 (RoB2) tool was used to assess the quality of appraisal in this SR. The RoB2 tool is a revised version of the original risk of bias tool that aimed to overcome certain limitations of the original version^38^. The RoB2 is structured according to five domains (randomization process, deviations from intended interventions, missing outcome data, measurement of the outcome, selection of the reported result), and overall bias. Judgment can be at “low” or “high risk” or can express “some concerns.” After two researchers independently assessed the risk of bias using the RoB2, they cross-checked the results and reached an agreement through discussions with each other about any disagreements or unclear judgments.

### Data extraction

The data were extracted and converted into an excel file based on the Cochrane recommendations^39^: publication year, country, study design, sample size of each group, participants’ characteristics including mean and range of age, contents of intervention, duration and frequency of intervention, and main outcomes.

The numerical values, such as the mean and standard deviation (SD), of the main outcomes (i.e., child night awakenings, maternal sleep quality, and maternal depression) were determined, as well as the sample size at certain time points after the intervention (within 1 to 4 months). The main outcome of sleep problems in children as recorded by their mothers in a sleep diary was event rate and the sample size at the aforementioned time points after the intervention.

### Data synthesis and statistical analysis

Mean and SD were used for continuous variables such as child night awakenings, maternal sleep quality, and depression scores. The odds ratio and 95% confidence interval were calculated as data synthesis statistics for dichotomous data, such as the child sleep problems for dichotomous data, such as the child sleep problems. For three of the studies^40–42^, an email was sent to the corresponding author of each in order to request for additional data. Additionally, 95% confidence intervals (CIs) were used in the calculation of the effect size of the overall mean and each effect size. Review Manager (RevMan) version 5.3 was used for meta-analysis. A homogeneity test between studies was performed using chi-square tests, and I^2^ tests. Meta-analysis using a random-effect model is appropriate for integrating intervention studies. These include different interventions, different results of ROB assessments and different participants’ characteristics^43–45^. Studies included in meta-analysis have heterogeneity. Therefore, all subgroup analyses were applied to the random-effect model.

## Results

### Selection of studies

**Figure 1:**
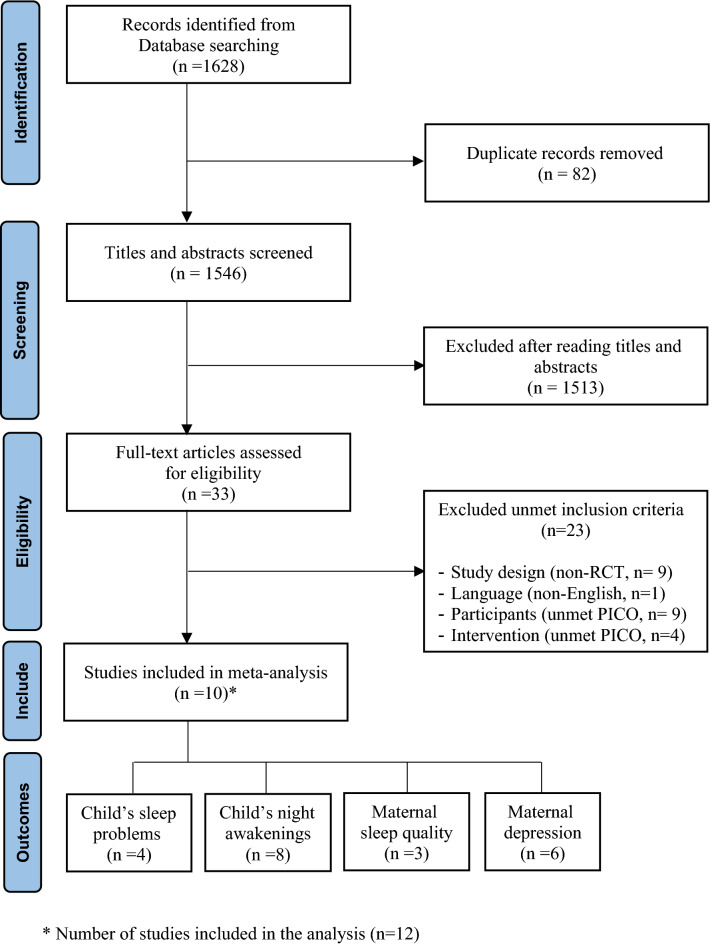
Flow chart of study selection.

The selection process of the studies was based on the PRISMA guidelines as illustrated in Fig. 1. A total of 1628 studies were initially searched: 644 studies from PubMed, 470 studies from Embase, 216 studies from CINHAL, and 298 studies from Cochrane. Of these, 82 studies were excluded due to duplication. Among the remaining 1546 studies, 1513 studies were excluded because they were not related to the PICO of this study as determined by the title and abstract of each study. After 33 full-text studies were reviewed, 23 studies were excluded according to specific exclusion criteria: non-English language (n = 1); irrelevant participants (n = 9); irrelevant intervention (n = 4); and non-RCTs design (n = 9), which included one mixed-method study, one second-data analysis, one study protocol, and six quasi-experimental designs using one group pre-post test. Finally, 10 studies were included in this study^24,30,31,40–42,46–49^. However, two of these studies were divided into two subgroups based on their participants and intervention type^41,47^; thus, a total of 12 subgroup analyses were included in the meta-analysis.

### Characteristics of included studies

The characteristics of the included studies are summarized in Table 1. The studies were published in 2002, 2007, 2009, 2011, 2013, 2017, 2018, and 2021, while two were published in 2016. Four studies were conducted in the United States, three studies in Australia, and one each in Canada, New Zealand, and Iran. The age of the children who participated in these studies ranged from 0 to 36 months. Regarding the intervention, five studies used settling methods, four studies used bedtime routines, and one study used both. In all the studies, the control group received usual care. The intervention periods were 2 weeks (three studies), 4 weeks, 8 weeks, 40 weeks, and from prenatal to 3 weeks after birth; the intervention period was not given in three studies. The time for assessing outcome measurements was up to 12 months after completing the intervention. Regarding the outcome variables, the presence of child sleep problems (two studies), the number of child night awakenings (eight studies), maternal depression measured by EPSD (three studies), and maternal sleep quality measured by PSQI (three studies) were used to evaluate the effect of BSIs on children.

**Table 1:** Characteristics of included studies.

Author (year), country	Study^a^ design	Range of Child age	Intervention group (n)	Control group (n)	Period of Intervention	Outcomes analyzed^b^	Results^c^
Galland (2017), New Zealand	RCT	Within 1 months	Settling methods (n = 192)	Usual care (n = 209)	2 sessions of training (1) Antenatal (2) 3 weeks postpartum	Child sleep problem (Yes or No)	↔
						Child night awakenings (Number)	↔
Galland (2017), New Zealand	RCT	Within 1 months	Settling methods (prevention of sleep problems) (n = 196)	Usual care (n = 209)	2 sessions of training (1) Antenatal (2) 3 weeks postpartum	Maternal depression (EPSD)^c^	↔
Gradisar (2016), Australia	RCT	6–16 months	Settling methods (n = 14)	Usual care (n = 14)	Not mentioned	Child night awakenings (Number)	↓
Hiscock (2002), Australia	RCT	6–12 months	Settling methods (n = 76)	Usual care (n = 76)	Not mentioned	Child sleep problem (Yes or No)	↓
						Maternal depression (EPSD)	↔
Hiscock (2007), Australia	RCT	7 month	Settling methods (n = 174)	Usual care (n = 154)	Not mentioned	Child sleep problem (Yes or No)	↓
						Maternal depression (EPSD)	↓
Mindell (2009), US	RCT	6–17 months	Bed time routine (n = 134)	Usual care (n = 72)	2 weeks	Child night awakenings (Number)	↓
Mindell (2009), US	RCT	18–36 months	Bed time routine (n = 133)	Usual care (n = 67)	2 weeks	Child night awakenings (Number)	↓
Mindell (2011), US	RCT	18–36 months	Bed time routine (n = 84)	Usual care (n = 84)	3 weeks	Child night awakenings (Number)	↓
						Maternal sleep quality (PSQI)	↑
Mindell (2018), US	RCT	3–18 months	Bed time routine (n = 64)	Usual care (n = 59)	2 weeks	Child night awakenings (Number)	↓
						Maternal sleep quality (PSQI)	↑
						Child sleep problem (Yes or No)	↓
						Maternal depression (EPSD)	↔
Paul et al. (2016), US	RCT	2 week after birth	Settling methods (n = 140)	Usual care (n = 139)	40 weeks (3, 16, 28, 40 weeks)	Child night awakenings (Number)	↔
Stremler (2013), Canada	RCT	2 week after birth	Settling + bedtime routine methods (n = 123)	Usual care (n = 123)	4 weeks	Child night awakenings (Number)	↔
						Maternal depression (EPSD)	↔
Rouzafzoon (2021), Iran	RCT	2–4 months	Bedtime routine methods (n = 41)	Usual care (n = 41)	8 weeks	Child night awakenings (Number)	↔
						Maternal sleep quality (PSQI)	↑
						Maternal depression (EPSD)	↓

^a^Study design: RCT, randomized controlled trial.

^b^Outcomes analyzed: EPSD, Edinburgh Postnatal Depression Scale; PSQI, Pittsburgh Sleep Quality Index.

^c^Results: ↑ or ↓: Statistically significant increase or decrease (*p*< 0.05); ↔ : no statistically significant changes (*p*< 0.05).

### Quality assessment of included studies

**Figure 2:**
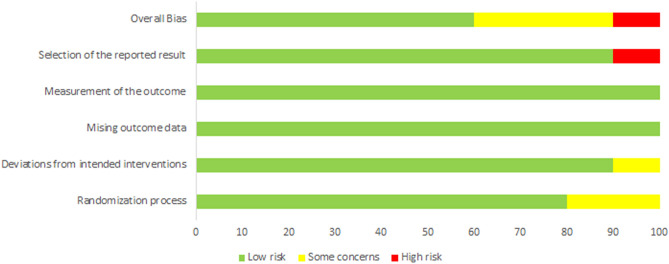
Risk of bias graph: based on researchers’ opinions about each risk of bias item, percentages are presented for all included studies.

The results of the quality assessment using RoB2 are presented in Fig. 2. and Table 2. The overall bias was “high risk” in one study, “some concerns” in three studies, and “low risk” in six studies. In the randomization process item, two studies were judged to have “some concerns” because they randomly assigned study participants and did not mention a specific allocation sequence or allocation concealment. Another study was classified under “some concerns” due to deviations from the intended interventions and because the study described the impossibility of blinding. The risk of bias in the selection of the reported results was assessed as “high risk” in another study because it reported the outcome based on meaningful results by means of converting categorical variables. However, using the other RoB2 evaluation criteria, all of the included studies were judged to be low risk.

**Table 2:** Risk of bias summary: researcher’s opinions bout each risk of bias item for all included studies.

First Author (Year)	Randomization process	Deviations from intended interventions	Missing out come data	Measurement of the outcome	Selection of the reported result	Overall
Galland (2017)	+	+	+	+	+	+
Gradisar (2016)	+	+	+	+	+	+
Hiscock (2002)	+	+	+	+	+	+
Hiscock (2007)	+	+	+	+	+	+
Mindell (2011)	?	+	+	+	+	?
Mindell (2009)	?	+	+	+	+	?
Mindell (2018)	+	+	+	+	+	+
Paul (2016)	+	+	+	+	−	−
Rouzafzoon (2021)	+	?	+	+	+	?
Stremler (2013)	+	+	+	+	+	+

+  = Low risk, ? = Some concerns, − = High risk.

### Effect of the behavioral sleep interventions on child sleep problems

**Figure 3:**
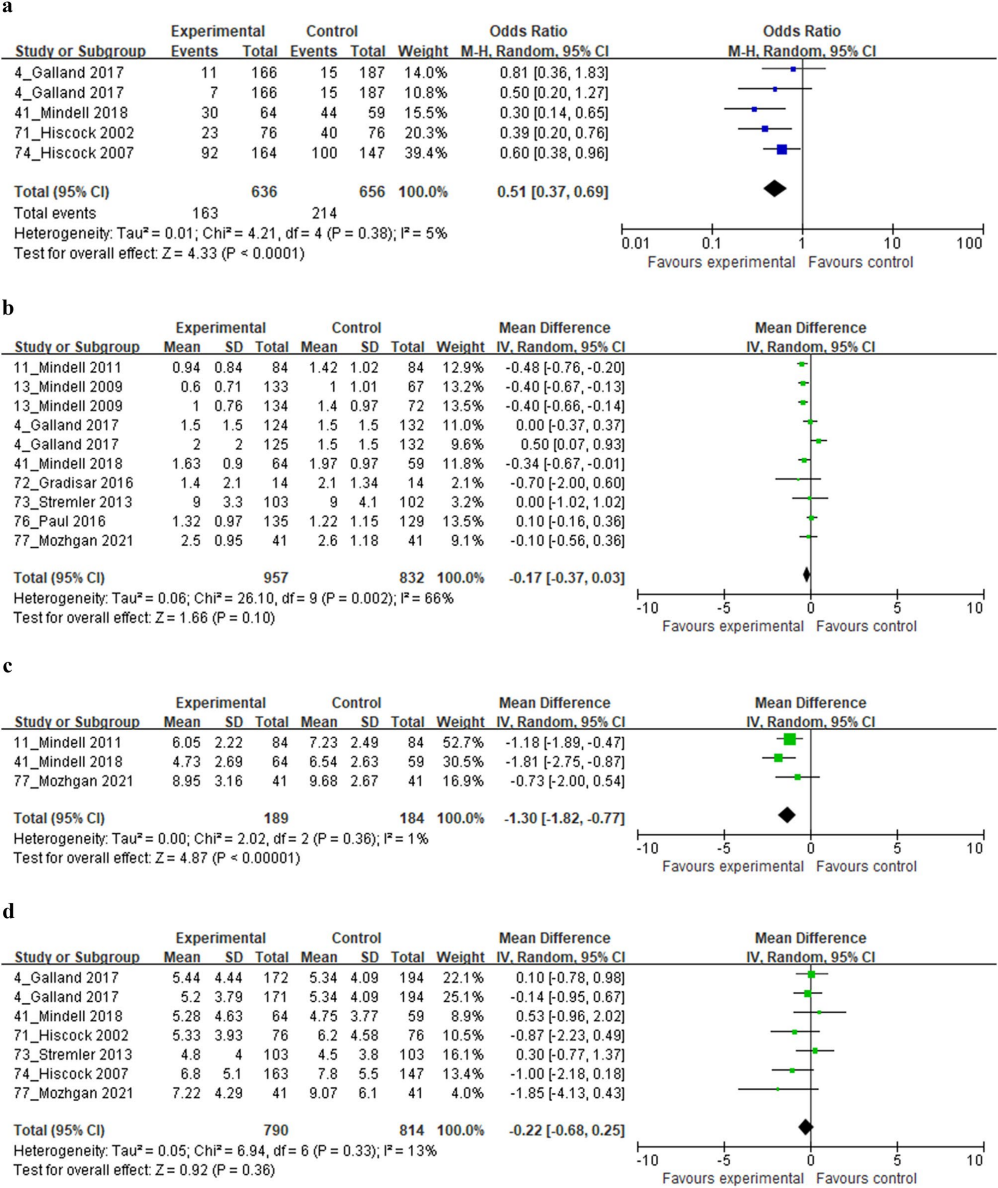
Forest plot comparing the effects of behavioral sleep intervention. (**a**) Child sleep problems reported by mothers, (**b**) Child night awakenings, (**c**) Maternal sleep quality, and (**d**) Maternal depression.

The effects of BSIs on child sleep problems are presented in Fig. 3a. Studies have reported the presence of child sleep problems in both the intervention and control groups even after BSIs. These studies were homogeneous with an I^2^ value of 5%. The odds ratio of child sleep problems was 0.51 (95% CI 0.37–0.69; *p* < 0.00001; k = 5), which showed that BSIs significantly reduced child sleep problems in the intervention group compared to the control group.

### Effect of the behavioral sleep interventions on the number of child night awakenings

The effect of BSIs on the number of child night awakenings is shown in Fig. 3b. Studies reported the number of night awakenings in both intervention and control groups after the BSIs and were heterogeneous with I^2^ = 66%. The mean difference in the number of child night awakenings was − 0.17 (95% CI − 0.37–0.03; *p* = 0.10; k = 10), which showed no significance between groups.

### Effect of the behavioral sleep interventions on the maternal sleep quality

The effects of BSIs on maternal sleep quality are shown in Fig. 3c. Studies have reported the maternal sleep quality of both the intervention and control groups using PSQI after BSIs. These studies were homogeneous with an I^2^ value of 1%. The mean difference in maternal sleep quality was − 1.30 (95% CI − 1.82 to − 0.77; *p* < 0.00001; k = 3), which indicates that the BSIs significantly improved the maternal sleep quality of the intervention group compared to the control group.

### Effect of the behavioral sleep interventions on maternal depression

The effect of BSIs on maternal depression is shown in Fig. 3d.

Studies reported maternal depression in both intervention and control groups using EPSD after BSIs and were homogeneous with I^2^ = 13%. The mean difference in maternal depression was − 0.22 (95% CI − 0.68–0.25, *p* = 0.33; k = 7), indicating that there was no significant difference between groups.

## Discussion

This study was conducted to examine the effects of BSIs on the sleep of children and mothers. As a result of the previous study search, 10 RCTs published from 2002 to 2021 were included, and significant effects of the BSIs on child sleep problems and maternal sleep quality were found.

In children’s aspects, when comparing BSIs with usual care, BSIs significantly decreased child sleep problems as reported by their parents. Parent-reported child sleep can provide integrative information on environmental and behavioral dimensions and is the most commonly used method for measuring children’s sleep^50,51^. Parent-reported problematic sleep is a strong predictor of sleep problems in children^41^. Child sleep problems vary according to developmental stage, are common in early childhood, and decline with age^52^. Williamson et al. examined sleep behaviors from birth to middle childhood using a longitudinal research design over a ten-year period. They found that reports of child sleep problems by caregivers were most prevalent in the infant stage and reduced with age, and the most common problem from infancy to seven years old was night awakening^53^. Focusing on infants and toddlers, Mindell et al. investigated the development of infant and toddler sleep patterns and found that infant sleep patterns more clearly develop at 5–6 months. That is, night-time sleep starts to integrate at 5 months and stabilizes more clearly at 6 months^54^. These changes in children, especially in infants’ sleep problems and patterns, are due to maturation of the circadian system. Sleep–wake homeostasis is related to multiple parenting and environmental factors, such as feeding, exposure to light, and sound^50^. Therefore, BSIs for infants should be considered at this critical time (i.e., 5–6 months after birth).

The duration of a behavioral sleep intervention in addition to the child’s developmental stage could affect sleep outcomes. That is, a subgroup analysis of the effect of BSIs by children’s age and intervention duration may be meaningful for detailed practical implications. However, the number of studies included in the current study was so small that subgroup analyses could not be performed. Therefore, more RCTs on children of varied developmental stages and with varied intervention durations are needed to further examine the effects of BSIs. With the accumulation of such RCT research, more specific and practical applications of BSIs can be expected.

The child night awakenings did not significantly change given the type of behavioral sleep intervention. A similar result was found in a SR and meta-analysis of infants aged under 12 months^37^. The number of night awakenings is one of the most commonly used methods for measuring sleep problems^55^, but night awakenings may not actually be sleep problems in children, especially infants. A possible interpretation of these results may be related to the participants’ developmental characteristics. That is, regardless of BSIs, night-time sleep awakenings could normally occur in infants due to feeding and diaper change^56,57^, and their immature circadian system. Moreover, parents of breastfed infants have reported more frequent night awakenings and shorter night-time sleep periods^58^. Among the studies included in the current study, there were eight studies used night awakenings as an outcome variable. Although seven studies of them included infants under 12 months of age, only two studies consider night feeding^49^ and feeding type^40^, and most of the studies did not fully consider participants’ developmental characteristics. Therefore, future research needs to consider how to measure infants’ night awakenings caused by sleep problems,s not by feeding or diaper change.

Although more studies on sleep have measured sleep patterns or problems using actigraphy in recent years, we used parent-reported child sleep as one of the outcome variables because most of the included studies utilized it. It is known that parent-reported sleep problems are easily measured, especially for infants and toddlers, but this method may overestimate sleep duration compared to actigraphy^51,59^. Actigraphy also has its limitations. For instance, it overestimates the number of awakenings since it can measure normal motor activity during sleep as a night awakening^60^. To consider the benefits and limitations of parent-reported data and actigraphy, the concurrent use of subjective and objective methods could be the best way to evaluate child sleep problems more accurately.

In mothers’ aspects, the current study found that maternal sleep quality significantly improved in the intervention groups. These results could be interpreted as sleep improvement in children through BSIs, and could also enhance maternal sleep quality. In previous studies, it was found that child sleep patterns have a significant correlation with maternal sleep. Covington et al.^61^ found that total maternal sleep time was associated with mothers’ perception of their toddlers’ sleep problems when sleeping with their toddlers. The number of night awakenings in infants was significantly positively correlated with that of mothers^62^. Goldman et al.^63^ examined the association between child and maternal sleep using actigraphy in typically developed children and children diagnosed with autism spectrum disorder. In this study, they found significant associations between time spent in bed, sleep time, and sleep fragmentation in mothers and children^63^.

In contrast, BSIs for children did not have a significant effect on maternal depression in the current study. This is inconsistent with the previous SR of psychosocial sleep interventions for children, which found a significant improvement in maternal depression^37^. Maternal depression could be related to their child’s sleep and the postpartum period. In a previous SR study of postpartum depression in healthy mothers, the overall prevalence of postpartum depression was approximately 12%^64^. Most postpartum depression is serious in the early postpartum period but decreases and becomes stable over time^65,66^. That is, because there were more mothers in their early postpartum period who were included in the present study than in the study of Kemplar et al.^35^, it seems that maternal depression might be related to the postpartum period, not the child’s sleep. In turn, this resulted in an insignificant effect of behavioral sleep intervention on maternal depression. Maternal postpartum depression could also be affected by various factors, such as history of anxiety or depression, prenatal depressive symptoms, and maternal fatigue^65–68^. Because of these other factors, maternal depression might not be improved by BSIs for their children.

In addition, the inappropriate use of EPDS for measuring maternal depression has been found in previous RCTs. EPDS was originally developed to measure maternal depression in the postpartum period^69^, which is defined as the period until 6 months after birth^70^. However, several studies included in the current study measured maternal depression using EPDS even if the mothers were not in the postpartum period^24,42,46^. Therefore, maternal depression might not be correctly assessed because of the use of inappropriate measurement tools. Therefore, more research is needed to measure maternal depression using appropriate instruments, and compounders affecting maternal depression should be considered when examining the effect of BSIs for children, on maternal depression. Additionally, future studies are needed to identify the long-term effects of BSIs on maternal depression and changes in maternal depression using a longitudinal study design.

As a result of the current study’s meta-analysis, BSIs, including settling and bedtime routine interventions, can reduce sleep issues in children and improve the mothers’ sleep quality. The application of age-appropriate planned sleep behavior interventions and sleep culture can contribute to the formation of positive sleep habits for both mother and child. It could contribute to early adaptation to the sleep environment and improvement of sleep quality. Therefore, the application of BSI can be implemented when the circadian rhythm is stabilized. Nurses should prepare the BSI plan and provide the mothers with the information soon after childbirth. An accuracy-evaluation of the number of night awakenings using an actigraph is required in future studies, and more studies measuring maternal depression or fatigue using appropriate tools are needed.

The current study has several limitations. First, only ten RCTs were included in the current study because of the limited number of RCTs on child BSIs. Because of the limited number of included studies, we could not perform subgroup analyses according to the ages of the children and the durations of the interventions, which could have helped provide detailed guidelines for clinical practice. Therefore, it is necessary to conduct more RCTs and accumulate evidence on the effects of child BSIs. Second, the current study could not use the data monitored by actigraphy because most studies confirmed sleep patterns using a sleep diary or a sleep-related questionnaire. However, in terms of measuring the sleep quality of children and adults, actigraphy could provide more objective data^71,72^; thus, future studies need to use actigraphy to achieve and evaluate outcomes of sleep intervention. Third, the current study did not separate participants according to developmental stage (e.g., infants, toddlers) because of the characteristics of the included studies. Since a child’s age could be a confounder of their sleep pattern, future RCTs need to consider child sleep patterns by developmental stage. Fourth, because the duration of interventions and follow-up times were different in most of the included studies, this could cause deviations in the meta-analysis results. Therefore, future SR and meta-analysis studies need to identify the effects of BSIs according to the duration of intervention and follow-up times. Fifth, because the sample size of the control group in Galland’s (2017) study was considered twice, some caution is needed not to overestimate results.

## Conclusion

This study found that BSIs in children significantly improved child sleep problems and maternal sleep quality. In general, RCTs of BSIs for children are limited, and therefore, more RCTs need to be conducted to provide further evidence for BSIs. Future SR and meta-analysis studies need to examine the effect of BSIs according to the child’s age, duration of intervention, and outcome measurement time points.

## Supplementary Information

Supplementary Information.

## Data Availability

The datasets used and/or analyzed during the current study are available from the corresponding author on reasonable request.
