# Supplementary material for: Effectiveness of behavioral sleep interventions on children’s and mothers’ sleep quality and maternal depression: a systematic review and meta-analysis
Source: Sci Rep. 2022 Mar 9;12:4172. doi: 10.1038/s41598-022-07762-8 (PMC8907206; doi:10.1038/s41598-022-07762-8)
Supplement: Supplementary file 1 — Supplementary Information. [file 41598_2022_7762_MOESM1_ESM.pdf]

Supplementary Table 1. Systematic literature review search terms and strategy

Search levels	Search terms
Search #1	“infant, newborn"[MeSH] OR infant OR newborn OR newborn infant OR baby OR child
Search #2	sleep[MeSH] OR sleep initiation and maintenance disorders[MeSH] OR initiation OR maintenance OR disorders OR insomnia OR sleep disturbance OR sleep problem OR wake OR night awaking OR unsettled OR settle OR settling OR bedtime problems OR actigraphy OR infant sleep
Search #3	behavior[MeSH] OR behavior OR therapeutics OR therapy OR treatment OR Behavior Therapy OR behavioral treatment OR Behavior Modification OR Behavior Modifications OR methods OR intervention OR interventions OR infant care OR child care
Search #4	mothers[MeSH] OR Care giver OR carer OR fatigue OR tiredness OR exhaustion OR burn-out OR maternal sleep OR maternal mood OR depression OR depressive disorders
Search #5	#1 AND #2 AND #3 AND #4
Filters: Humans, Randomized controls trials	

Supplementary Table 2. All the extracted variables

Outcome			Variables included in Meta-analysis
**Child**			
	Sleep	Parent reported sleep problem (yes or no)	O
		Number of night wakings	O
		Brief infant sleep questionnaire (BISQ)<br>Children's sleep habits questionnaire (CSHQ)<br>Maternal Cognitions about Infant Sleep Questionnaire (MCISQ)<br>Child Sleep Question from the Longitudinal Study of Australian Children (LSAC)	
	Mood	Child mental health (Child Behavior Checklist)	
		25-item strengths and difficulties questionnaire (SDQ)<br>salivary cortisol(stress)	
	Behavior	Parent Behavior Checklist	
		sleep-related behaviors	
		Child behavior checklist 1½ -5 year old	
	QoL	PedsQL4.0	
**Mothers**			
	Sleep	PSQI	O
		Epworth Sleepiness Scale (ESS)	
		Maternal nocturnal (9pm to 9 am) sleep<br>General sleep disturbance scale (GSDS)	
	Mood	EPSD	O
		Profile of mood states (POMS)	
		Depression subscale score (DASS)	
		BMIS (Brief mood introspection scale)	
		STAI maternal anxiety (State-trait anxiery (STAI), Form Y)	
		SF12: mental health scores	
	Parenting	Maternal parenting styles(11 items from the Longitudinal Study of Australian Children)<br>Parent stress index (PSI)	
		Parenting behavior checklist	
		Fatigue VAS	
